# Median Nerve Entrapment after Supracondylar Humeral Fracture in a Young Child

**DOI:** 10.1155/2019/1543126

**Published:** 2019-10-30

**Authors:** C. Granier, E. Maury, B. Coulet, M. Delpont, J. Cottalorda, C. Sleth, R. Carré, D. Louahem

**Affiliations:** Department of Pediatric Orthopedic Surgery, Lapeyronie Hospital, University of Montpellier, 371 Avenue Doyen Gaston Giraud, 34090 Montpellier, France

## Abstract

Median nerve entrapment after supracondylar humeral fracture in children is rare. We report a case of Gartland type III supracondylar humeral fracture complicated by an entrapment of the median nerve following closed reduction and percutaneous pinning in a 5-year-old child. The diagnosis of entrapment was made 14 months post injury following progressive motor and sensory palsy. Resection and end-to-end suture were performed, leading to complete sensory and motor recovery eight months later. This nerve complication is often unnoticed and should be suspected systematically before and after reduction of all displaced supracondylar humeral fracture in children. The indication of resection-suture or nerve graft depends on the entrapment and the delay of the palsy.

## 1. Introduction

Gartland type III supracondylar humeral fractures (SCHF) in children are known for their severe neurovascular complications. Extension-type fractures with posterolateral displacement are the most frequent and responsible for nerve or neurovascular injuries with a frequency of 9% to 17% [1–10]. The injury of the median nerve and particularly the anterior interosseous nerve (AIN) are the most frequently involved [1, 8, 10–13]. Its entrapment within the fracture site is rare and often left unnoticed especially in younger children. The diagnosis is delayed by 3 months to 2.5 years after the trauma and is characterized by progressive sensory-motor palsy [14]. Electromyogram (EMG), ultrasound, and MRI imaging are powerful tools to help in the confirmation of the nerve injury and its entrapment within the fracture site or the callus. The treatment is based on surgical exploration and neurolysis or end-to-end anastomosis or nerve graft. We report a case of a progressive sensory-motor palsy by entrapment of the median nerve within the fracture callus after type III SCHF in a 5-year-old child. The diagnosis and treatment of similar cases are discussed in light of the fairly scarce literature.

## 2. Case Report

**Figure 1:**
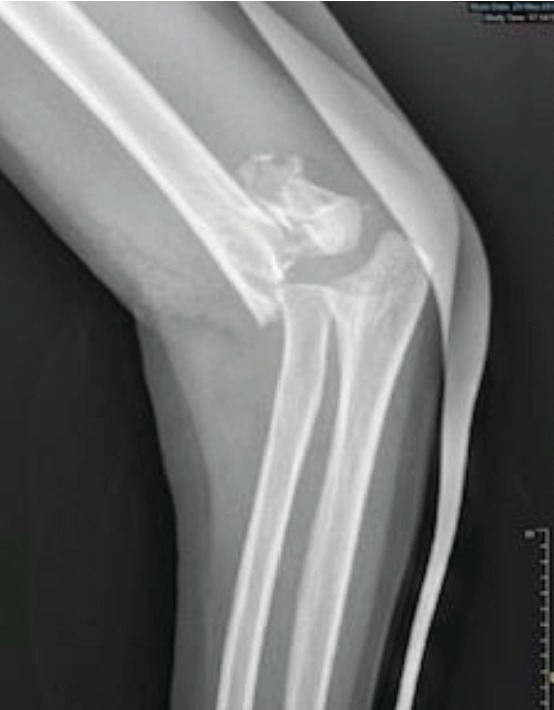
Gartland type III SCHF.

**Figure 2:**
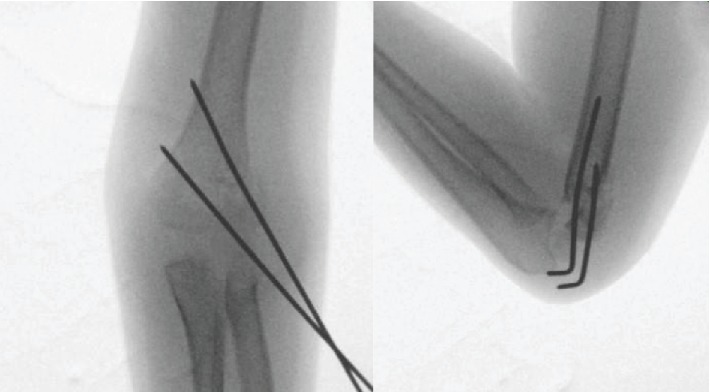
Lateral percutaneous pinning: front and side view.

**Figure 3:**
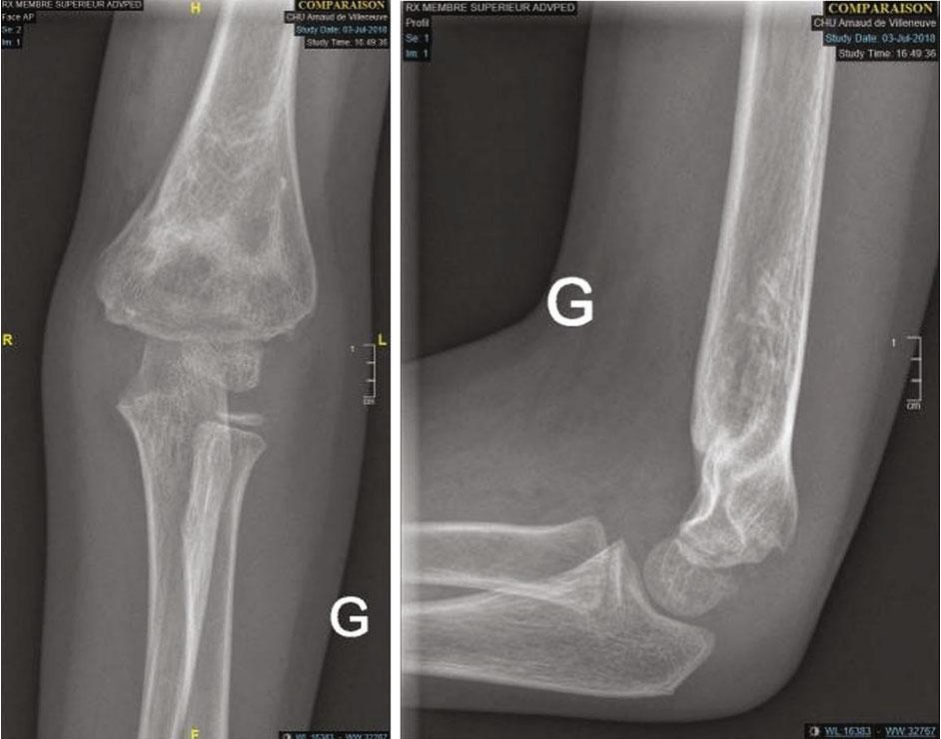
Complete remodeling of the distal end of the humerus: 6 months postoperatively—front and side view.

The patient is a 5-year-old right-handed child, admitted to the pediatric emergency room for trauma of the left elbow after falling from a 1.5 meter height. Initial clinical examination found a deformity of the elbow with swelling, ecchymosis in the anterolateral side, and absence of the radial pulse but with a well perfused hand. Neurological examination was very difficult because of the child's young age and the painful trauma. Radiographic examination of the elbow revealed a Gartland type III SCHF with posterolateral displacement (Figure 1). Closed reduction of the fracture and its fixation by percutaneous lateral pinning were urgently performed (Figure 2). Postoperative X-ray control showed an imperfect reduction with moderate posterior translation but with no inter-fragment gap that could have suspected an entrapment of the median nerve. The return of the normal radial pulse occurred two hours postoperatively. The healing fracture occurred one month later, followed by removal of the pins. Total remodeling of the distal humeral end was radiologically noticed six months later (Figure 3).

**Figure 4:**
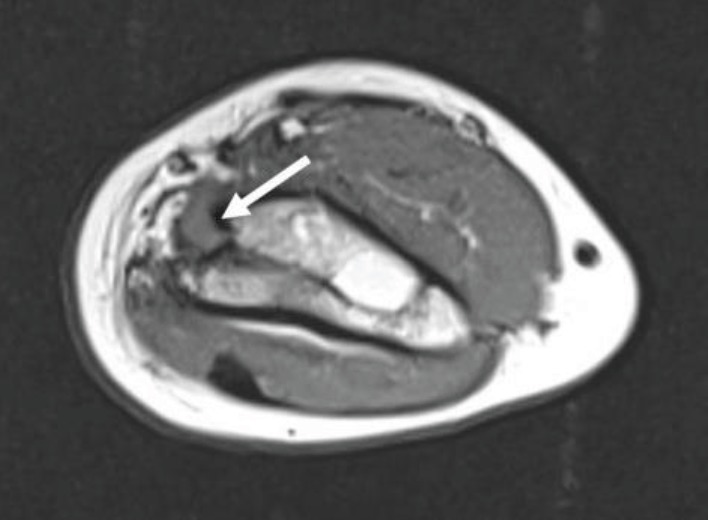
T1 MRI sequence showing median nerve entrapment upstream of the bone callus (white arrow).

**Figure 5:**
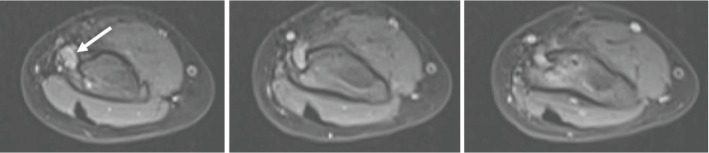
T2 DPFS MRI sequence showing hypertrophy (white arrow) and median nerve entrapment within the consolidated fracture.

One year and two months later, the parents noted that their child had a functional discomfort when using the left hand and an amyotrophy of the thenar eminence and the forearm. Clinical examination found sensory-motor deficit in the median nerve with hypoesthesia and palsy of both the thumb opposition and AIN. Electroneuromyography confirmed severe truncal injury of the median nerve with an advanced denervation pattern of the abductor pollicis brevis muscle; the topography was in favor of a lesion nerve at the healed fracture site. Ultrasonography of the elbow revealed an entrapment of the nerve within the fracture callus. The median nerve was thickened (0.56 cm of diameter) entering the bone callus; invisible downstream along a 1.5 cm, it reappeared at the level of the elbow joint space with a normal diameter (0.18 cm). MRI confirmed the entrapment of the median nerve within the callus (Figures 4 and 5) with signs of nervous suffering as hypertrophy (0.5 cm of diameter against 0.2 cm at the same location on the contralateral side), hypersignal on DP fat sat sequences, and amyotrophy of the flexor, pronator quadratus, and pronator teres muscles.

**Figure 6:**
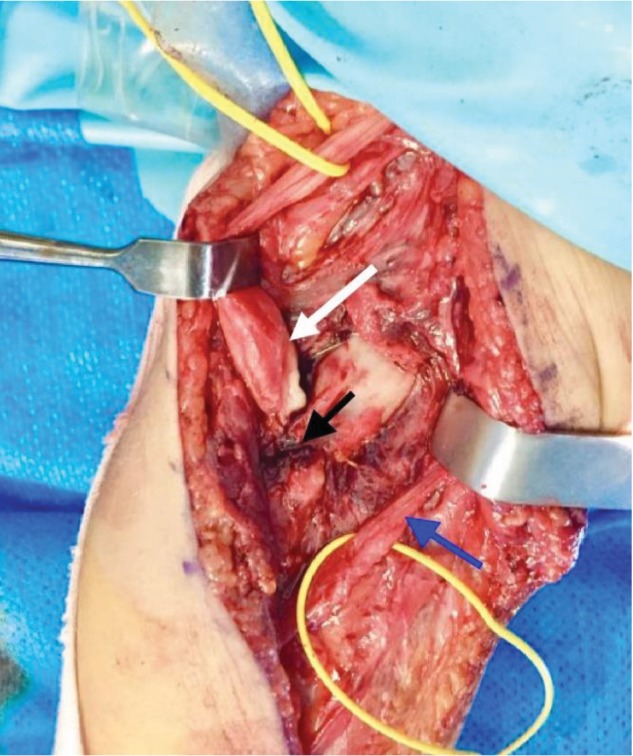
Entrapment of the hypertrophic proximal end of the median nerve (white arrow) within the consolidated fracture (black arrow) and fibrosis with atrophy of the distal end of the median nerve (blue arrow).

**Figure 7:**
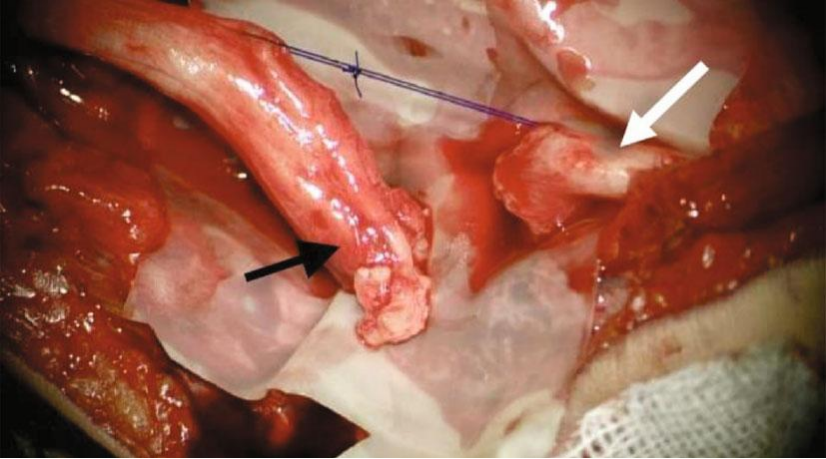
Post resection view flush of the bone callus of the proximal end (black arrow) and of the atrophic fibrosis part along a 1.5 cm section next to the distal end (white arrow) of the median nerve.

**Figure 8:**
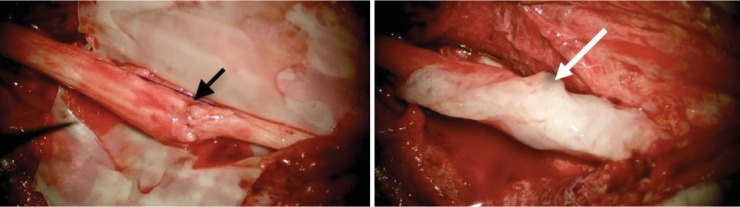
Terminoterminal direct type suture of the median nerve (black arrow) reinforced with biological sealant (white arrow).

Surgical exploration and dissection under a microscope following an anterior Henry approach revealed the presence of substantial fibrosis in the anteromedial side of the elbow facing the fracture callus. After careful dissecting and excision of the fibrosis, the proximal end of the median nerve was greatly thickened and entrapped within the bone callus. Its atrophic distal end was sheathed within a fibrosis facing the callus along a 1.5 cm section (Figure 6). The resected proximal end at the level of the callus and the regulation of the distal end by excision of all atrophic segment allowed for direct end-to-end suture, added to biological glue; the elbow was flexed at 60° to protect the suture from any tension (Figures 7 and 8). Postoperative immobilization was insured by a posterior splint for three weeks. Rehabilitation was performed by a physiotherapist specializing in the upper limb until complete recovery.

Follow-up at four months postoperatively showed normal mobility of the elbow and a travelling Tinel sign 3.5 cm below the elbow, demonstrating nerve regeneration. Partial motor recovery of the AIN was observed through the return of the distal interphalangeal joint flexion and the improvement of the thumb-index pinch. Eight months postoperatively, motor and sensory recovery was complete and amyotrophy of the thenar eminence and the forearm had disappeared. At the latest follow-up of twelve months, total return function was observed and the child could use normally again his left hand without discomfort or dysesthesia.

## 3. Discussion

Neurological complications in extension-type III SCHF in children represent 15% of cases [9], most of them involving damage of the median nerve and the AIN [1, 7, 10, 11]. Posterolateral displacement is generally incriminated [1]. These complications are often unnoticed, and their immediate postoperative diagnosis is difficult, particularly in young children [14]. It is most often neurapraxia, and spontaneous nerve recovery is ruled [1]. Entrapment of the median nerve within the fracture callus is exceptional in children. In 1974, Post and Haskell [15] published a case of median nerve entrapment after a displaced SCHF with an absence of radial pulse and complete motor palsy of the median nerve in a 5-year-old child. Three months after closed reduction and percutaneous pinning, persistence of the complete palsy was confirmed by electromyography. Surgical exploration revealed the entrapment of the median nerve within the callus. Nerve resection-reconstruction was followed by complete recovery. In 1986, Karlsson et al. [16] reported 4 cases of SCHF with entrapment of both brachial artery and median nerve within the fracture site. Nerve extrication and neurolysis allowed for complete recovery. In 1988, Thorleifsson et al. [17] described a case of entrapment of the median nerve after closed reduction of a displaced SCHF with acute injury of the median nerve. The absence of nerve recovery after ten weeks required surgical exploration confirming the nerve entrapment within the fracture callus. Dissection and neurolysis under a microscope allowed complete recovery of the nerve function. In 2006, Louahem et al. [1] reported 3 (11%) cases of entrapment of both brachial artery and median nerve within the fracture site among 28 cases of acute median nerve injuries, whether isolated or associated with brachial artery damage, in a series of 210 type III SCHF in children. In 2016, they compiled 3 (9%) other identical cases of neurovascular entrapment among 32 cases of median nerve lesions, associated with vascular injuries in a series of 404 type III SCHF in children [2]. In all these 6 cases, the entrapment diagnosis was established during the surgical exploration imposed by an imperfect closed reduction of the fracture and preoperative ischemia (white hand). Immediate extrication and neurolysis of the median nerve allowed for complete nerve recovery after a 3-month postoperative period. In 2014, Metineren et al. [18] reported a case of entrapment of the median nerve in the bone callus after a SCHF with acute palsy of the AIN in a 7-year-old child, initially treated by surgical reduction and pinning. The absence of recovery 6 months postoperatively needed surgical exploration, hence confirming entrapment of the nerve within the healed fracture site. After resection of the bone callus, the nerve was covered by fibrosis around the fracture site. Resection with the regulation of the nerve ends was followed by end-to-end anastomosis using Tisseel Fibrin Glue. Complete nerve and thumb opposition recovery was observed 12 months postoperatively.

The early diagnosis of the median nerve entrapment would reduce morbidity in such cases. The challenge of performing an urgent thorough examination of the neurological status, particularly in young children with type III SCHF, is responsible for the most part of diagnosis delay. Posterolateral displacement, vascular injury, imperfect reduction, and postreduction interfragment gap must encourage the search for median nerve entrapment [1, 2]. Repeated neurological examination postoperatively is crucial. The occurrence of dysesthesia, pain exacerbation, and motor or sensory deficit must require complementary investigations. Ultrasounds and especially MRI imaging reveal the course of the median nerve and signs of neuropathy and can also serve as an adjunct to electromyography in the evaluation of muscle denervation through the assessment of muscle atrophy. They allow to perform an early diagnosis of entrapment or lesion of the median nerve and thus for early, noninvasive treatment. In such cases, early extrication associated with neurolysis of the median nerve leads to complete and fast nerve recovery [1, 2, 16].

## 4. Conclusion

Median nerve entrapment after displaced SCHF in children remains rare but must not be underestimated, particularly in young children. Advances in noninvasive imagery, ultrasonography, and particularly MRI allow for an early and even preoperative diagnosis of entrapment within the fracture site and for rapid intervention by simple surgical exploration and neurolysis. At a later stage, resection-end-to-end suture of the nerve, when possible, may allow for complete recovery in a young child. Other surgical procedures reserved for old entrapment with extended nerve injuries such as nerve resection-graft do not always allow for a complete recovery, requiring further palliative surgery by tendinous transfer.

## Supplementary Materials

Supplementary Materials The video is titled: “Preoperative video showing median nerve entrapment.” Description of the video: “This preoperative video shows, after dissection of the front side of elbow, the entrapment of the hypertrophic proximal end of the median nerve within the consolidated fracture and fibrosis with atrophy of the distal end of the median nerve. The flush resection of the bone callus of the proximal end and of the atrophic fibrosis part along a 1.5 cm section next to the distal end of the nerve allowed a terminoterminal type suture of the elbow flexed at 60°.”
